# Identification of Hydroxyl and Polysiloxane Compounds via Infrared Absorption Spectroscopy with Targeted Noise Analysis

**DOI:** 10.3390/polym17111533

**Published:** 2025-05-30

**Authors:** Kuang-Yuan Hsiao, Ren-Jei Chung, Pi-Pai Chang, Teh-Hua Tsai

**Affiliations:** Department of Chemical Engineering and Biotechnology, National Taipei University of Technology, No. 1, Section 3, Zhongxiao E. Road., Da’an District, Taipei 106344, Taiwan

**Keywords:** FTIR spectroscopy, hydroxyl functional groups, polysiloxane, alcohol solvents, spectral noise analysis, hydrogen bonding, polyvinyl butyral, solvent compatibility

## Abstract

This investigation of hydroxyl and polysiloxane absorption peaks in elastic polymer composites reveals significant spectral shifts within the fingerprint region of FTIR spectra. Using poly(vinyl butyral) (PVB) as the base polymer and poly(vinyl acetate) (PVAc) and poly(vinyl alcohol) (PVA) as reference materials, solvent effects on polymer–solvent interactions were systematically analyzed. Among the tested alcohol solvents, PEG 400 induced the most pronounced spectral changes, with the C=O stretching band shifting from 1740 to 1732 cm^−1^ and the O–H band significantly broadening and downshifting to around 3300 cm^−1^, reflecting strong hydrogen-bonding interactions. Wavelet-based noise reduction effectively enhanced the signal-to-noise ratio, reducing the baseline standard deviation by over 90%. This study introduces a novel noise-enhanced FTIR recognition model that integrates baseline noise metrics to improve detection sensitivity. The model successfully uncovers subtle structural variations in polymer–solvent systems that are typically masked by conventional FTIR techniques, advancing materials analysis and providing a robust framework for future FTIR-based diagnostics and material characterization.

## 1. Introduction

FTIR spectroscopy is a well-established analytical technique used to characterize the molecular structure of organic and polymeric materials [1]. It enables the rapid identification of functional groups based on characteristic absorption bands, especially within the fingerprint region (1500–600 cm^−1^), where complex vibrational interactions reflect subtle structural differences among compounds [2,3]. In recent years, the analysis of spectral noise—traditionally viewed as interference–has emerged as a novel approach for enhancing spectral resolution and recognizing solvent-induced effects in composite systems [4].

PVB, polyethylene glycol (PEG), and siloxane-based materials are commonly used in flexible electronic devices, adhesives, and coatings due to their tunable mechanical and chemical properties [5]. These polymers contain hydroxyl, carbonyl, and ether functional groups, whose vibrational signatures are sensitive to environmental polarity and hydrogen-bonding interactions [6]. Among these, alcohol-based solvents such as ethanol, n-propanol, n-butanol, and PEG 400 are particularly known to modulate these interactions, resulting in spectral shifts and peak broadening in FTIR spectra [7].

However, existing FTIR studies primarily focus on peak identification and intensity comparisons, often overlooking subtle yet informative variations in baseline fluctuations and low-intensity regions [8]. These spectral “noise signatures”, particularly within the low-energy fingerprint range, can offer valuable insights into intermolecular interactions, solvent compatibility, and hydrogen-bonding mechanisms [9]. The lack of standardized methods for interpreting such noise characteristics represents a gap in current spectroscopic analysis.

This investigation of the FTIR spectral responses of PVB-based polymer composites subjected to various alcohol solvents reveals characteristic changes. Emphasis is placed on spectral shifts, hydrogen-bonding effects, and the emergence of distinctive noise features in the low-wavenumber region. By comparing control and treated samples, this work identifies solvent-specific interactions with hydroxyl and carbonyl groups and introduces a noise-enhanced spectral recognition model for hydroxyl-containing polymers. These findings provide a new analytical framework for characterizing polymer–solvent compatibility and advancing FTIR-based materials analysis. In contrast to traditional FTIR analysis, which relies solely on peak intensity and position, this study integrates spectral noise indicators—specifically, baseline standard deviation—as diagnostic features. This noise-enhanced FTIR recognition model enables the detection of subtle structural differences that are typically masked by random noise or solvent interference. The noise-enhanced FTIR recognition model incorporates baseline noise standard deviation as a diagnostic metric to distinguish subtle spectral variations that are often masked in conventional FTIR analyses.

## 2. Materials and Methods

### 2.1. Materials

Poly(vinyl acetate) (PVAc), poly(vinyl butyral) (PVB), polyethylene glycol (PEG 400), and polysiloxane resin were used as base materials. Solvents, including ethyl acetate (EtOAc), ethanol (5% aqueous), n-propanol, n-butanol, acetone, and toluene, were all of analytical grade and used without further purification. Custom-synthesized poly(vinyl alcohol) (PVA) was prepared in-house to verify the intermediate transformation between PVAc and PVB in the FTIR pathway.

The detailed sample preparation methods and experimental designs are described in Section 2.2.

### 2.2. Sample Preparation

To investigate the influence of different additives on FTIR absorption characteristics, three types of polymer samples were prepared using a saturated aliphatic hydrocarbon polymer as the base. The samples were formulated and processed under controlled temperature (50 °C), stirring, and solvent evaporation to ensure homogeneity and minimize phase separation.

**Figure 1:**
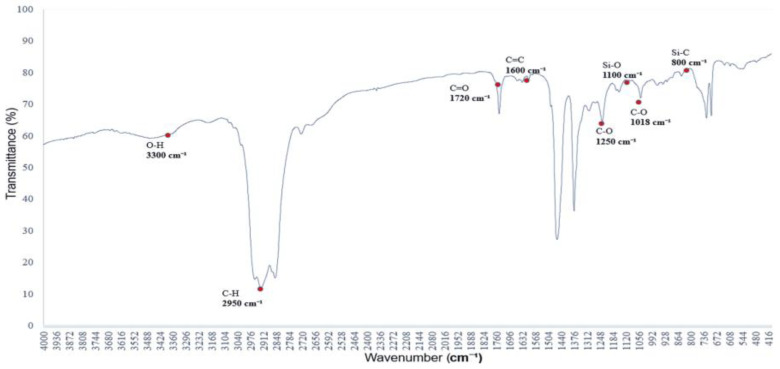
FTIR spectrum of a saturated alkane polymer dissolved in ethyl acetate: C–H and functional group vibrational characteristics.

(1) Control sample (Figure 1):

The base polymer dissolved in ethyl acetate (EtOAc) was stirred at 50 °C. This formulation serves as the unmodified control for FTIR baseline comparison.

**Figure 2:**
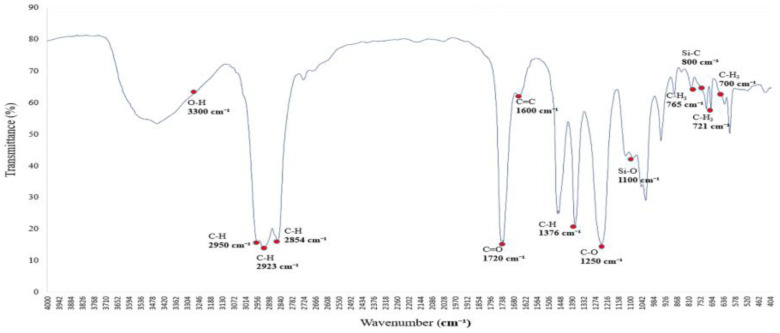
FTIR spectrum of the polymer blended with miscible PVAc: characteristic absorption peaks of C=O and C–O groups.

(2) PVAc-modified sample (Figure 2):

The base polymer + PVAc was dissolved in EtOAc and stirred at 50 °C. This was carried out to evaluate the influence of PVAc on the C=O stretching region.

**Figure 3:**
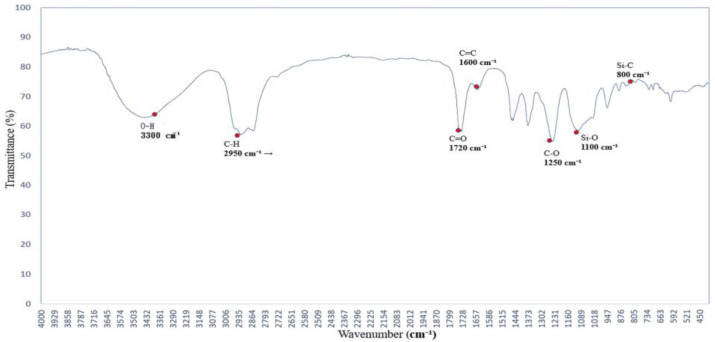
FTIR spectrum of the polymer blended with polyethylene glycol (PEG 400), showing absorption bands near 3300 cm^−1^ (O–H stretching) and at 1250 and 1100 cm^−1^ (C–O–C ether linkages). These bands indicate hydrogen-bonding capability and confirm the structural features of PEG.

(3) PEG-modified sample with ethanol (Figure 3):

Base polymer + PVAc + PEG 400 + ethanol (5% water) was co-dissolved in EtOAc and stirred at 50 °C. This was carried out to explore PEG-induced shifts in O–H and C–O–C regions.

All formulations were stabilized before FTIR scanning. The resulting spectra were analyzed to identify vibrational shifts related to additive incorporation and spectral improvement following noise reduction.

### 2.3. FTIR Spectroscopy

FTIR spectroscopy was performed using a Nicolet iS50 spectrometer equipped with an attenuated total reflectance (ATR) instrument. Spectra were collected in the 4000–500 cm^−1^ range with a resolution of 4 cm^−1^ and 32 scans per sample. Background spectra were recorded before each measurement and automatically subtracted. Each sample was scanned at least three times to ensure data reproducibility.

### 2.4. Spectral Analysis

All infrared spectra were collected using OMNIC software (version not specified) with standard baseline correction applied prior to peak assignment and interpretation. No advanced frequency-domain processing (such as FFT or wavelet analysis) was performed in this study.

This ensured that all peak assignments were based on raw spectra with minimal preprocessing, preserving the interpretative integrity of hydrogen bonding and solvent interaction effects.

### 2.5. Data Comparison and Interpretation

FTIR spectra from different sample groups were systematically compared. Variations in the C=O, O–H, and C–O bands were analyzed to determine the influence of solvent polarity, hydrogen bonding, and molecular interactions. A custom reference table of functional group absorption frequencies was constructed to summarize solvent-specific shifts and intensity variations. Tables and figures were generated to illustrate correlations between material composition and FTIR spectral characteristics.

## 3. Results

### 3.1. Baseline FTIR Characteristics of Alkane-Based Adsorbent Surfaces

Figure 1 presents the infrared absorption spectrum of a saturated alkane polymer dissolved in ethyl acetate. The spectrum displays eight major absorption bands, with peak wavenumbers located at (01) 2954 cm^−1^, (02) 2923 cm^−1^, (03) 2854 cm^−1^, (04) 1463 cm^−1^, (05) 1376 cm^−1^, (06) 765 cm^−1^, (07) 721 cm^−1^, and (08) 700 cm^−1^.

For example, the bands in the range of 2950–2850 cm^−1^ correspond to C–H stretching vibrations, while those in the range of 1465–1340 cm^−1^ are attributed to CH₂ and CH₃ bending vibrations, which are characteristic of alkyl group structures [10].

**Figure 4:**
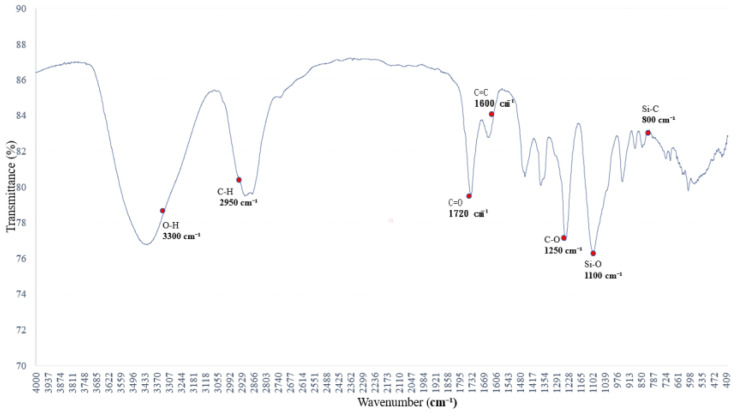
FTIR spectrum of the sample from Figure 3 further blended with 5% aqueous ethanol: variations in hydrogen bonding and C–O absorption bands.

To facilitate subsequent analysis, the characteristic absorption peak positions of the saturated alkane polymer dissolved in ethyl acetate (ethyl acetate/EtOAc), as shown in Figure 1, are summarized in Table 1. These peaks correspond to the vibrational modes of the C–H, CH₂, and CH₃ functional groups [11]. These absorption peaks are consistent with those of saturated alkanes, confirming the primary composition of the sample. Furthermore, Figure 2, Figure 3 and Figure 4 present the infrared absorption spectra of saturated alkane polymers blended with various miscible substances, providing a basis for further investigation of their structural and physicochemical properties.

### 3.2. Graphite’s Characteristic Equation

Figure 2 shows the infrared absorption spectrum of a saturated alkane polymer dissolved in ethyl acetate (ethyl acetate/EtOAc) and blended with miscible PVAc [12]. Compared to Figure 1, the infrared absorption spectrum exhibits not only the original eight major peaks but also several additional absorption bands, with corresponding wavenumbers as follows: (1) 3300 cm^−1^, (2) 2950–2850 cm^−1^, (3) 1727 cm^−1^, (4) 1465–1340 cm^−1^, (5) 1376 cm^−1^, (6) 1250 cm^−1^, (7) 1096 cm^−1^, and (8) 1018 cm^−1^.

Among them, the peak at 1727 cm^−1^ corresponds to the stretching vibration of the carbonyl (C=O) group, confirming the presence of the PVAc structure [13]. In addition, the peak at 1363 cm^−1^ is attributed to the CH₂ group, while the peaks at 1223 cm^−1^ and 1018 cm^−1^ are assigned to C–O stretching vibrations, which are characteristic absorption features of the PVAc molecular structure [14]. These absorption peaks are distinctive features of the infrared spectrum of PVAc, confirming the structural influence of PVAc [15].

### 3.3. Hydroxyl Group Dynamics Under PEG 400 Exposure: FTIR Insights

The FTIR spectrum of polyethylene glycol (PEG 400), shown in Figure 3, reveals characteristic absorption peaks associated with both O–H stretching and C–O–C ether linkages [16,17], reflecting its hydrogen-bonding potential and molecular architecture.

The broad absorption band centered around 3300 cm^−1^ is attributed to O–H stretching vibrations, indicating strong hydrogen-bonding interactions. This feature suggests that PEG 400 actively participates in hydrogen bond formation with surrounding molecules. Additional bands are observed at 2950 and 2870 cm^−1^ (C–H stretching), 1720 cm^−1^ (C=O stretching), and 1465–1340 cm^−1^ (C–H bending).

Of particular interest are the C–O–C stretching vibrations at ~1250 and 1100 cm^−1^, which serve as molecular fingerprints of PEG’s ether structure. These peaks confirm the presence of ether linkages and highlight PEG’s high polarity and hydrophilicity, contributing to its solvent interaction capabilities.

Together, these spectral characteristics establish a reliable reference for evaluating PEG’s role as a hydrogen bond donor and structural modifier in polymer systems. This foundational profile supports its application in FTIR-based analyses of surface chemistry, compatibility, and solvation effects [18,19].

### 3.4. Hydrogen-Bonding Perturbations in PVB Under Aqueous Ethanol Adsorption

Figure 4 presents the FTIR spectrum of the saturated alkane polymer blended with ethyl acetate, PVAc, and 5% aqueous ethanol.

Polyethylene glycol (PEG 400) at 4.78 wt% exhibits characteristic absorption peaks in the FTIR spectrum associated with 5% aqueous ethanol, primarily reflecting vibrational changes in O–H and C–O bonds. The leading absorption bands are observed at (1) 3500–3200 cm^−1^, (2) 2980 and 2900 cm^−1^, (3) 1465 and 1375 cm^−1^, (4) 1250 and 1100 cm^−1^, and (5) 950–900 cm^−1^.

The FTIR spectral features of PEG are predominantly influenced by the presence of O–H and C–O–C functional groups [20]. The O–H stretching absorption band of ethanol, observed in the 3500–3200 cm^−1^ range, indicates pronounced hydrogen bonding. As the water content increases, this absorption band becomes broader and shifts in wavenumber [21].

### 3.5. Polysiloxane–PVB Interfacial Interactions: Spectral Interpretation

**Figure 5:**
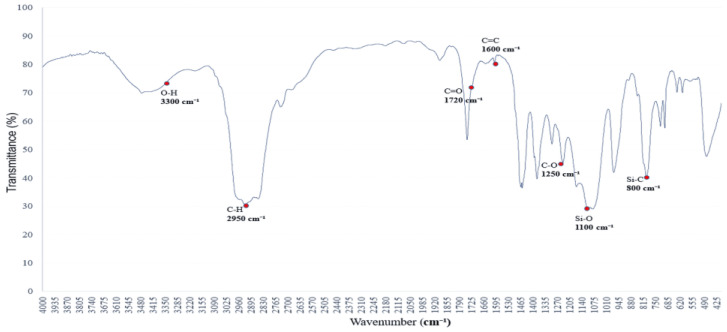
FTIR spectrum of PVAc and polysiloxane resin blend.

**Figure 6:**
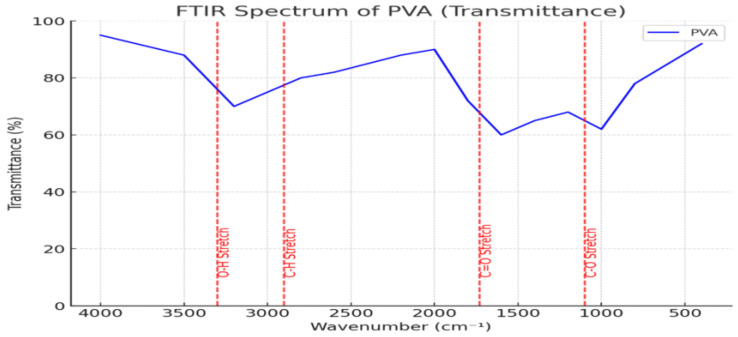
The FTIR spectrum of the synthesized PVA sample shows characteristic absorption peaks corresponding to O–H, C–H, and C–O stretching vibrations. These features serve as a reference for structural comparison with PVB during the transformation from PVAc.

Figure 5 and Figure 6 show the spectra of PVB mixed with siloxane-based polymers and additional solvents. The absorption peaks at 1250–1100 cm^−1^ and 800–750 cm^−1^ correspond to Si–O–Si and Si–C vibrations, respectively.

These bands confirm the presence of polysiloxane networks and suggest a relatively stable spectral profile compared to highly polar systems.

### 3.6. Comparative Solvent Effects on Surface Functional Groups: Spectral Signatures Targeted Noise Reduction for Enhanced FTIR Signal Clarity in Adsorbent Surface Analysis

#### 3.6.1. FTIR Reference Spectrum of Synthesized PVA for Structural Transformation Analysis

To investigate the structural transformation of PVAc to PVB via PVA as an intermediate, a custom-synthesized PVA sample was analyzed using FTIR spectroscopy [22]. The resulting spectrum served as a key reference for identifying functional groups and understanding their role in the transformation process [22,23].

The FTIR spectrum of PVA revealed a broad O–H stretching vibration band at ~3200–3500 cm^1^, indicating extensive hydrogen bonding between hydroxyl groups [24]. A C–H stretching vibration near ~2900 cm^1^ and a sharp C–O absorption band around ~1000–1100 cm^1^ were also observed, representing typical structural features of PVA [24,25]. Moderate C=O absorption near ~1700 cm^1^ suggests slight oxidative modification, useful for comparative analysis with PVB [23,24].

As shown in Figure 6, these characteristic peaks provide a spectral basis for tracking hydroxyl group retention and transformation during the PVAc → PVA → PVB conversion. Subsequent analysis with the PVB spectrum enables an evaluation of hydrogen bond evolution and copolymer interaction mechanisms.

#### 3.6.2. Comparative Effects of PEG 400 and PVA on PVB: Spectral and Hydrogen-Bonding Insights

To evaluate how different hydroxyl-containing agents influence the hydrogen-bonding environment and structural stability of PVB, a comparative FTIR analysis was conducted using PEG 400, PVA, and ethanol [26,27]. These agents vary in molecular weight, polarity, and hydrogen-bonding capacity [28].

**Figure 7:**
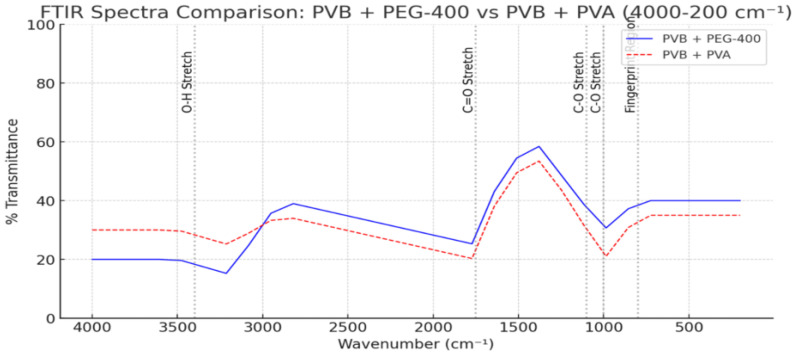
FTIR spectra of PVB blended with PEG 400 and PVA, showing variations in O–H and C=O stretching regions. The differences indicate distinct hydrogen-bonding interactions induced by each additive.

The FTIR spectra revealed that PEG 400 induces significant broadening in the O–H stretching region (~3300 cm^−1^), along with notable downshifts in the C=O (~1732 cm^−1^) and C–O (~1075 cm^−1^) absorption bands. These shifts reflect extensive intermolecular hydrogen-bonding and plasticization effects. PVA, in contrast, causes sharper but smaller shifts in the same regions, suggesting more localized interactions through structural integration [29]. As shown in Figure 7, PVB samples modified with PEG 400 and PVA exhibited distinct absorption patterns in the O–H and C=O regions, confirming the differing hydrogen-bonding interactions induced by these additives [24].

**Figure 8:**
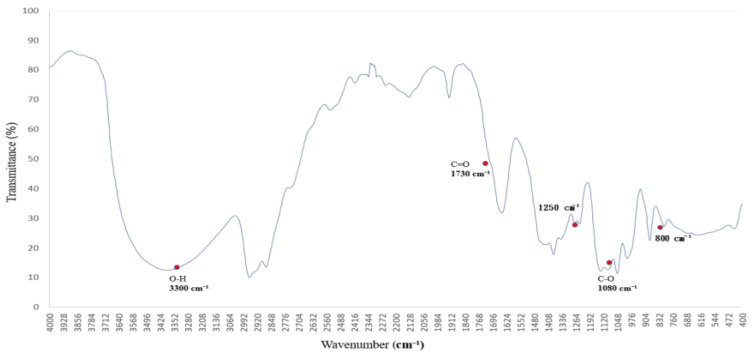
FTIR spectrum of PVB in aqueous ethanol (5% water), exhibiting a broadened O–H band and slight shifts in C=O and C–O regions due to temporary hydrogen bonding and dipolar interactions with the solvent.

To provide a solvent-based contrast, ethanol (containing 5% water) was also evaluated [22]. Figure 8 displays the spectral response of PVB in aqueous ethanol, revealing a broadened O–H absorption band and minor shifts in the C=O and C–O regions. These changes are attributed to temporary hydrogen bonding and polarity-induced dipole interactions rather than permanent structural alteration [28].

Together, Figure 7 and Figure 8 illustrate how different hydrogen-bonding sources—from polymeric additives to small polar solvents—modulate the FTIR signatures of PVB. PEG 400 demonstrates the strongest impact on the hydrogen-bonding network, followed by ethanol and PVA. These findings highlight the importance of additive polarity and molecular flexibility in tuning polymer compatibility and spectral behavior [26].

#### 3.6.3. Impact of n-Propanol on PVB Functional Group Vibrations

To assess the interaction between *n*-propanol and PVB, FTIR spectroscopy was employed to monitor changes in key functional groups. The addition of *n*-propanol caused broadening of the O–H stretching band (~3200–3400 cm^−1^), suggesting disruption or rearrangement of hydrogen bonding in the PVB matrix. A moderate downshift in the C=O stretching peak (from ~1740 to ~1734 cm^−1^) indicated dipole–dipole interactions and potential solvent penetration into the polymer structure.

In addition, the C–O stretching band (~1080 cm^−1^) showed slight shifts and changes in intensity, likely due to the polarization of ether linkages within PVB. The low-frequency region (<1500 cm^−1^) exhibited increased background noise and additional minor bands, reflecting changes in intermolecular interactions and structural disorder induced by *n*-propanol.

**Figure 9:**
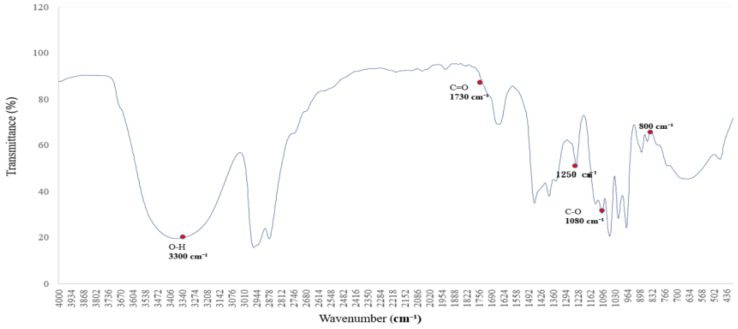
FTIR absorption spectrum of PVB interacting with *n*-propanol, showing variations in O–H, C=O, and C–O stretching regions indicative of hydrogen bonding and solvent–polymer interactions.

As shown in Figure 9, these spectral changes confirm that *n*-propanol influences both the hydrogen-bonding network and the carbonyl environment of PVB. Compared to ethanol, *n*-propanol induces less pronounced but still significant variations, which can be attributed to its lower dipole moment and larger molecular size. These findings suggest that *n*-propanol offers moderate interaction strength and partial structural reversibility upon evaporation, providing practical guidance for solvent selection in PVB material design.

#### 3.6.4. Interactions of PVB with n-Propanol: Spectral Implications

To evaluate the solvent-dependent spectral responses of PVB, FTIR analysis was conducted using four alcohol-based solvents: ethanol, *n*-propanol, *n*-butanol, and PEG 400 [30]. Among these, *n*-butanol exhibited the weakest interaction with PVB, as evidenced by minimal shifts and narrow peaks in key functional group regions [31].

**Figure 10:**
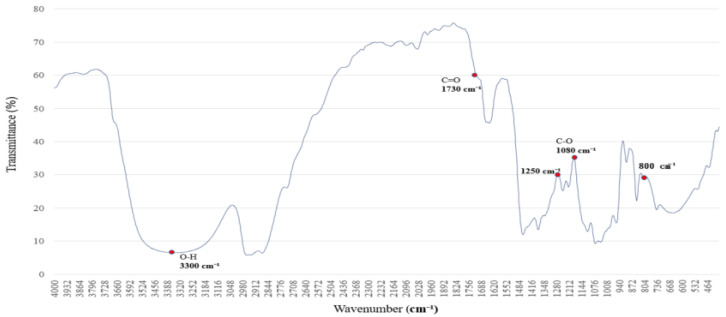
FTIR absorption spectra of PVB interacting with *n-butanol*, showing weak hydrogen bonding with minimal peak shifts in O–H, C=O, and C–O regions.

As shown in Figure 10, the O–H stretching vibration (~3200–3400 cm^−1^) in PVB/*n*-butanol appeared at ~3310 cm^−1^ with negligible broadening, indicating weak hydrogen bonding. Similarly, the C=O stretching band shifted slightly from 1740 to 1737 cm^−1^ with only a ~6% intensity increase, and the C–O region (1085 → 1082 cm^−1^) showed minor variation. These small spectral shifts are attributed to *n*-butanol’s low polarity, reduced dipole interaction, and limited molecular adsorption due to its longer carbon chain. Additionally, no significant background signals were observed in the low-energy region (<1500 cm^−1^), confirming minimal structural perturbation.

To synthesize these findings, Table 2 and Table 3 summarize the characteristic shifts in C=O, C–O, and O–H bands across all solvent conditions. PEG 400 induced the most pronounced changes, particularly in the O–H band (~3300 cm^−1^) and C=O region (~1732 cm^−1^), highlighting strong hydrogen bonding and dipole–dipole interactions. Ethanol and *n*-propanol had moderate effects, while n-butanol had the least influence.

This solvent dependency reveals a trend: PEG 400 > ethanol > *n*-propanol > *n*-butanol, corresponding to decreasing polarity and hydrogen-bonding capacity. These spectral variations not only indicate differences in molecular-level interactions but also provide insight into selecting solvents for optimizing PVB’s mechanical stability, flexibility, or hydrophobicity, depending on the application.

### 3.7. Targeted Noise Reduction for Enhanced FTIR Signal Clarity in Adsorbent Surface Analysis

#### 3.7.1. Sources of Spectral Noise in FTIR Analysis of Adsorbent Materials

Accurate FTIR analysis of adsorbent materials requires clear identification of characteristic functional groups [36]. However, polyethylene glycol (PEG 400), widely used for surface modification, introduces various spectral noise sources due to its high hydroxyl content and hygroscopic nature. These factors complicate peak assignment and reduce signal clarity.

When interacting with PVB, PEG 400 enhances intermolecular hydrogen bonding, resulting in broadened O–H bands (~3300 cm^−1^) and downshifted C=O (~1740 → 1732 cm^−1^) and C–O (~1085 → 1075 cm^−1^) absorptions. While these shifts confirm strong dipole–dipole and hydrogen-bonding interactions, they also increase background interference, especially in the low-wavenumber region (<1500 cm^−1^), where water retention and matrix heterogeneity can cause additional spurious bands.

**Figure 11:**
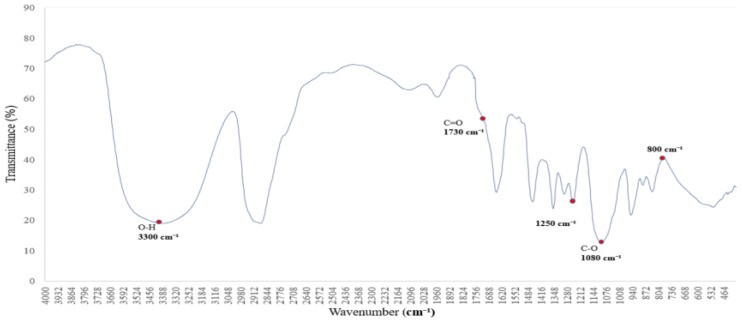
FTIR spectrum of PVB blended with polyethylene glycol (PEG 400), showing broad O–H bands and enhanced C=O/C–O absorptions, with noticeable low-wavenumber noise due to PEG’s hygroscopic properties.

As shown in Figure 11, the FTIR spectrum of PVB/PEG 400 displays overlapping absorptions and baseline instability, particularly in the 1000–1500 cm^−1^ region. Such spectral noise complicates the interpretation of surface-bound functional groups and may lead to overestimation of bond strength or misidentification of minor peaks [37]. These issues highlight the need for targeted preprocessing techniques, such as baseline correction and moisture compensation, to restore spectral resolution and ensure accurate characterization in high-humidity or polar solvent environments [38].

#### 3.7.2. Practical Implications and Future Integration of Noise Reduction in Surface Analysis

The application of wavelet-based denoising in FTIR analysis has shown, in the related literature, significant potential to enhance the interpretability of spectra from surface-functionalized polymers such as PVB. These methods can suppress high-wavenumber noise, reduce baseline drift, and improve peak clarity—particularly for functional groups such as O–H and C=O, which are often susceptible to environmental interference and instrument variability [39].

Although this study did not directly implement automated noise reduction techniques, the integration of such preprocessing tools—especially wavelet transform algorithms—into future FTIR pipelines could be valuable. By improving the signal-to-noise ratio (SNR) and preserving key vibrational features, these methods may facilitate the more reliable detection of solvent-dependent spectral changes and surface functional group interactions.

Looking forward, automated denoising approaches may also enable large-scale data analysis and pattern recognition through advanced statistical tools and machine learning. This advancement holds strong potential for in situ diagnostics, material compatibility screening, and the real-time monitoring of chemical changes in adsorbent systems.

### 3.8. Summary: Solvent–Surface Functional Group Interactions and Adsorption Implications

This section summarizes how different alcohol-based solvents affect PVB’s FTIR spectra. PEG 400 showed the strongest interaction, causing significant shifts in the C=O, O–H, and C–O bands due to its high polarity and hydrogen-bonding ability. In contrast, n-butanol caused minimal spectral changes, suggesting weaker polymer–solvent interactions. Ethanol and n-propanol exhibited intermediate effects.

These results demonstrate that FTIR spectral shifts—and potentially noise-related features—can be used to assess solvent compatibility and hydrogen-bonding strength. The fingerprint region (<1500 cm^−1^) may contain subtle indicators of intermolecular interactions or solvation effects.

In future studies, incorporating environmental sensitivity and baseline fluctuation metrics into FTIR analysis could enhance functional group detection and support broader material screening strategies.

### 3.9. Statistical Analysis of FTIR Spectra

Although statistical testing was not applied in this study, future work may benefit from incorporating statistical methods to enhance the analytical rigor of FTIR spectral interpretation—especially in cases involving subtle vibrational shifts or low-intensity spectral features.

For example, paired-sample *t*-tests, standard deviation analysis, and effect size estimations (e.g., Cohen’s d) could be employed to quantitatively assess the impact of preprocessing techniques such as wavelet denoising or baseline correction. These statistical tools would allow researchers to validate improvements in the signal-to-noise ratio (SNR), baseline stability, and peak resolution, especially in the fingerprint region, where noise interference is more pronounced.

Furthermore, as FTIR datasets become increasingly complex—often involving multiple solvents, additives, or functionalized materials—integrating statistical modeling with spectroscopic data may support multivariate analysis, chemometric classification, or even machine learning applications. These approaches could uncover latent patterns and functional group correlations that are not readily observable through traditional peak analysis alone.

In summary, the application of statistical frameworks in FTIR spectroscopy holds strong potential to improve spectral reliability, facilitate reproducibility, and support advanced surface characterization in polymer–solvent systems.

## 4. Conclusions

This study systematically investigated the infrared absorption behaviors of PVB composites in the presence of various alcohol-based solvents using FTIR spectroscopy. The analysis focused on vibrational changes in C=O, O–H, and C–O functional groups, as well as the appearance of solvent-induced baseline irregularities in the fingerprint region.

The key conclusions are as follows:

1. Solvent polarity and functional group sensitivity: Alcohol solvents significantly influenced the FTIR spectra of PVB by modulating hydrogen bonding and dipole–dipole interactions. Solvent polarity showed a clear correlation with peak broadening and vibrational shift magnitude. PEG 400 induced the most significant changes, likely due to its high polarity and strong hydrogen-bonding capacity.
2. Hydrogen-bonding behavior: The broadening and red-shifting of O–H absorption peaks under different solvent conditions confirmed the modulation of hydrogen bonding within the composite matrix. PEG 400 showed the strongest hydrogen-bonding interaction, followed by ethanol and n-propanol; n-butanol exhibited minimal effects.
3. Fingerprint region characteristics: Solvents with higher hydrophilicity (e.g., ethanol, PEG 400) introduced notable spectral variations in the low-energy fingerprint region (1500–600 cm^−1^). These variations may result from water absorption, microstructural fluctuations, or dynamic polymer–solvent interactions.
4. Potential of spectral preprocessing: Although this study did not implement noise reduction algorithms, future work may explore the use of wavelet-based preprocessing to improve signal clarity, especially in high-noise spectral zones. Such techniques could enhance peak identification in functionalized polymers.
5. Material design implications: The findings emphasize the importance of solvent selection when tuning polymer–solvent interactions for coatings, sensors, or flexible electronics. Systems enhanced with PEG 400 may offer greater hydrophilicity and functional sensitivity, while n-butanol-based systems may provide better structural stability for environments requiring lower reactivity.

In summary, this work highlights that FTIR spectroscopy is a powerful tool for monitoring solvent-induced vibrational behavior in polymers. Future studies may explore the integration of statistical tools or AI-assisted noise analysis to advance functional group detection and deepen our understanding of molecular-level interactions in polymeric materials.

## Figures and Tables

**Table 1:** Characteristic absorption peak positions of the saturated alkane polymer.

Vibrational Modes of Functional Groups	Absorption Peak Position/cm^−1^	Characteristics of Absorption Peaks
ν_C–H_	2950~2850	Doublet (alkyl group)
δ_C–H_	1465~1340	Broad peak (methyl/methylene group)
δ_CH3_	1380	Sharp peak (methyl group)

**Table 2:** Variation in characteristic absorption peak positions of C=O, C–O, and O–H groups in PVB under different alcohol-based solvents. Shifts to lower wavenumbers indicate the extent of dipole and hydrogen-bonding interactions.

Functional Group	Absorption Position (cm^−1^)	Shifted Absorption Position Under Alcoholic Solvents	Spectral Characteristics
C=O (carbonyl)	1740	Ethanol: 1736 (shifted to lower wavenumber) n-Propanol: 1734 (shifted to lower wavenumber) n-Butanol: 1737 (shifted to lower wavenumber) PEG 400: 1732 (shifted to lower wavenumber)	The carbonyl group exhibits strong dipole–dipole interactions with solvents. Increased polarity enhances hydrogen bonding, causing a downward shift in the absorption frequency [32,33].
C–O (ether)	1085	Ethanol: 1080 (shifted to lower wavenumber) n-Propanol: 1078 (shifted to lower wavenumber) n-Butanol: 1082 (shifted to lower wavenumber) PEG 400: 1075 (shifted to lower wavenumber)	PEG 400 contains abundant ether linkages, which enhance intermolecular interactions with PVB, resulting in more pronounced shifts to lower absorption frequencies [34].
O–H (hydroxyl)	3610–3670	Ethanol: 3298 (shifted to lower wavenumber) n-Propanol: 3305 (shifted to lower wavenumber) n-Butanol: 3310 (shifted to lower wavenumber) PEG 400: 3300 (shifted to lower wavenumber)	O–H stretching vibrations show notable broadening and a downward shift in wavenumber, suggesting strong hydrogen-bonding interactions [33].
C–H (alkyl)	2960	-	C–H stretching vibrations show no obvious changes under different solvents [34,35]

**Table 3:** FTIR absorption peak shifts of PVB under various alcohol solvent conditions. Data illustrate solvent-dependent interactions with functional groups, with PEG 400 causing the strongest and *n*-butanol causing the weakest effects.

Solvent Condition	C=O Stretching (cm^−1^)	O–H Stretching (cm^−1^)	C–O Stretching (cm^−1^)
PVB (control)	1740	3332	1085
PVB + Ethanol	1736 (peak shifted to lower wavenumber)	3298 (shifted to lower wavenumber)	1080 (shifted to lower wavenumber)
PVB + n-Propanol	1734 (shifted to lower wavenumber)	3305 (shifted to lower wavenumber)	1078 (shifted to lower wavenumber)
PVB + n-Butanol	1737 (shifted to lower wavenumber)	3310 (shifted to lower wavenumber)	1082 (shifted to lower wavenumber)
PVB + PEG 400	1732 (shifted to lower wavenumber)	3300 (shifted to lower wavenumber)	1075 (shifted to lower wavenumber)

## Data Availability

Data are contained within the article. Further inquiries can be directed to the corresponding author.
